# Metabolic control of gene transcription in non-alcoholic fatty liver disease: the role of the epigenome

**DOI:** 10.1186/s13148-019-0702-5

**Published:** 2019-07-18

**Authors:** Matthew C. Sinton, David C. Hay, Amanda J. Drake

**Affiliations:** 1 University/British Heart Foundation Centre for Cardiovascular Science, The Queen’s Medical Research Institute, University of Edinburgh, 47 Little France Crescent, Edinburgh, EH16 4TJ UK; 2 MRC Centre for Regenerative Medicine, University of Edinburgh, Edinburgh, EH16 4UU UK

**Keywords:** Non-alcoholic fatty liver disease, Mitochondria, TCA cycle, Alpha-ketoglutarate-dependent dioxygenases, Metabolite

## Abstract

Non-alcoholic fatty liver disease (NAFLD) is estimated to affect 24% of the global adult population. NAFLD is a major risk factor for the development of cirrhosis and hepatocellular carcinoma, as well as being strongly associated with type 2 diabetes and cardiovascular disease. It has been proposed that up to 88% of obese adults have NAFLD, and with global obesity rates increasing, this disease is set to become even more prevalent. Despite intense research in this field, the molecular processes underlying the pathology of NAFLD remain poorly understood. Hepatic intracellular lipid accumulation may lead to dysregulated tricarboxylic acid (TCA) cycle activity and associated alterations in metabolite levels. The TCA cycle metabolites alpha-ketoglutarate, succinate and fumarate are allosteric regulators of the alpha-ketoglutarate-dependent dioxygenase family of enzymes. The enzymes within this family have multiple targets, including DNA and chromatin, and thus may be capable of modulating gene transcription in response to intracellular lipid accumulation through alteration of the epigenome. In this review, we discuss what is currently understood in the field and suggest areas for future research which may lead to the development of novel preventative or therapeutic interventions for NAFLD.

## Background

Globally, non-alcoholic fatty liver disease (NAFLD) is the most common form of chronic liver disease [1], and comprises a spectrum of pathologies, from benign hepatic steatosis to inflammatory non-alcoholic steatohepatitis (NASH), liver cirrhosis, and hepatocellular carcinoma (HCC) [2]. Benign hepatic steatosis is the most common form of NAFLD and is characterised by a hepatic fat content of greater than 5% of total liver weight [3]. Approximately 37% of adult patients with benign steatosis will develop NASH, which is strongly associated with hepatic inflammation, insulin resistance (IR), and type 2 diabetes (T2D) [4]. Despite much research aimed at developing therapeutics to target the disease, currently, the only way to reverse the development of benign steatosis or NASH is through lifestyle modifications associated with weight loss, including diet and exercise [5], or gastric banding [6], for example, in a 5-year human study gastric banding was shown to be effective in reversing hepatic steatosis, NASH, and IR [6]. However this remains an invasive surgical procedure, the risks of which are exacerbated in overweight individuals [7]; indeed a systematic review of the outcomes found that a subset of patients experienced new or worsening features of NAFLD, including fibrosis, demonstrating that this surgery is not suitable in all cases [8]. A major hurdle in the development of therapeutics for NAFLD is that the cause is multi-factorial [9]. Early studies advanced a two-hit model, in which benign steatosis, characterised by the accumulation of intracellular triglycerides, represents the first ‘hit’. The second hit involves the additional presence of oxidative stress, the production of inflammatory cytokines and mitochondrial dysfunction [10]. This has now been further developed into a ‘multiple-hit’ model, which considers that numerous other factors may drive pathology, including diet and genetic predisposition [11].

## Obesity, type 2 diabetes, and NAFLD

The relationship between NAFLD, IR, and T2D is complex, and although NAFLD and its progression are strongly associated with the metabolic syndrome, it also occurs in the absence of overt IR and T2D. Indeed, epidemiological studies suggest that the presence of NAFLD may be predictive of the development of IR and T2D [3], but the precise molecular bases of these associations are not fully understood.

Hepatic steatosis is a consequence of an imbalance between hepatic lipid uptake, de novo lipogenesis, and lipid clearance. In obesity, the excess accumulation of lipids in visceral adipose tissue results in an increased release of free fatty acids (FFAs) into the circulation, with FFAs derived from adipose lipolysis being the principal source of lipids supplied to the liver [12]. In the steady state, stimulation of hepatic insulin receptors activates the PKB/AKT pathway, inducing phosphorylation and nuclear exclusion of the fork-head box protein (FOXO1) transcription factor [13]. In the presence of excess FFAs, insulin receptor activation is blunted, leading to a disruption of the insulin signalling pathway [14]. This disruption prevents the phosphorylation of FOXO1, allowing it to translocate to the nucleus, where it stimulates the expression of genes associated with gluconeogenesis and de novo lipogenesis [15]. This can stimulate hepatic intracellular lipid accumulation and the development of large lipid droplets within the cytoplasm [16]. Overnutrition, accompanied by hyperinsulinemia and hyperglycemia, drives steatosis by promoting de novo lipogenesis in the liver which contributes substantially to the accumulation of multiple lipid species [17]. Studies in rodents have shown that obesity is associated with an increase in the expression of lipogenic transcription factors, including carbohydrate response element binding protein (ChREBP) and sterol regulatory element binding protein-1c (SREBP-1c). This leads to a downstream increase in the expression of lipogenic genes, increased flux through acetyl-CoA carboxylase, and increased hepatic malonyl-CoA. These changes result in increased hepatic de novo lipogenesis, suppressed hepatic fatty acid oxidation, and the development of steatosis [18]. Further, the increase in hepatic diacylglycerol, which binds to and activates protein kinase C [19], can in turn inhibit the hepatic insulin signalling cascade [20]. FFAs also repress the expression of glucose transporter 4 (GLUT4), and studies in immortalised primary human hepatocytes suggest that the subsequent increase in intracellular glucose levels can stimulate glucokinase activity, providing more substrates for glycolysis through conversion of glucose to glucose-6-phosphate [21] and further contributing to de novo lipogenesis [22]. Increased rates of glycolysis may also provide pyruvate, which can be used as a substrate for the TCA cycle.

## Mitochondria and the TCA cycle in NAFLD

**Fig. 1:**
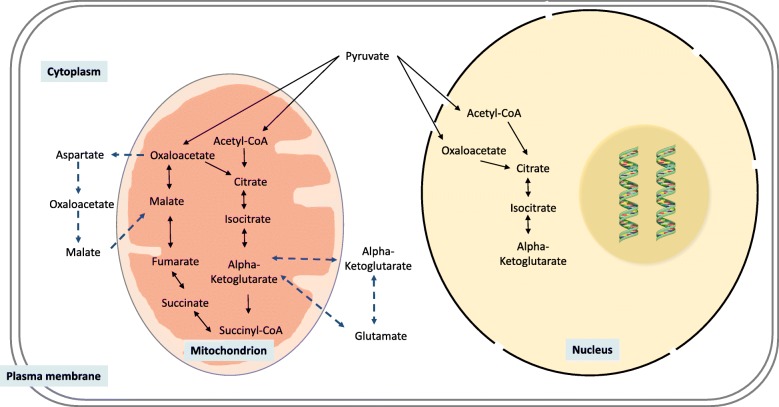
The TCA cycle occurs predominantly in the mitochondrial matrix, although some reactions can occur beyond the mitochondria, including in the cytoplasm and nucleus. Solid arrows represent the reaction direction (enzymes omitted for simplicity), whilst dashed arrows represent the transport of metabolites into the cytoplasm

Studies in humans and mice indicate that alterations in mitochondrial TCA cycle activity may play a central role in the pathogenesis of NAFLD [23, 24]. The TCA cycle occurs predominantly in the mitochondrial matrix and is a central pathway for the metabolism of amino acids, fatty acids, and carbohydrates, in order to generate cellular energy [25]. The cycle utilises the substrate acetyl coenzyme A (acetyl-CoA), which is generated by glycolysis via pyruvate, through the action of pyruvate dehydrogenase (PDH) [26] and through amino acid degradation and β-oxidation. Acetyl-CoA is oxidised through a series of steps, producing NADH and FADH_2_, which transfer electrons to the electron transport chain to fuel the production of ATP. Pyruvate can also be carboxylated by pyruvate carboxylase (PC) to form oxaloacetate (OAA), which then undergoes a condensation reaction with acetyl-CoA to form citrate (Fig. 1). Additionally, the TCA cycle can be fuelled by the deamination of glutamate by glutamate dehydrogenase, to produce α-ketoglutarate (αKG) [27]. Increased influx of substrates into the TCA cycle can increase cycle flux, as well as the abundance of the intermediates αKG, succinate, and fumarate [28].

Whilst the TCA cycle is typically a feature of the mitochondria, specific TCA cycle reactions can also occur in the cytoplasm [29] and the nucleus [30] (Fig. 1). For example, αKG can be generated in the cytoplasm by glutamate dehydrogenase, before being transported into the mitochondria. The malate/aspartate shuttle exports aspartate to the cytoplasm, where it is converted to oxaloacetate, and then to malate, whilst also oxidising NADH to NAD^+^. Malate and NAD^+^ are then transported back into the mitochondria, and αKG is simultaneously exported to the cytoplasm. Data from studies in PC12 cells suggest that alterations in the balance of NADH and NAD^+^ may play a role in the development of fatty liver, through increased oxidative stress [31] and disruption of β-oxidation, leading to increased lipid accumulation [32]. There are mitochondrial and cytoplasmic forms of the TCA cycle enzymes isocitrate dehydrogenase (IDH) and malate dehydrogenase (MDH). Fumarate hydratase, which only exists in one form, is most commonly located in the mitochondria but can be found in the cytoplasm and can translocate to the nucleus upon DNA damage [33]. Furthermore, several mitochondrial TCA cycle enzymes, including PDH, PC, and isocitrate dehydrogenase 3 (IDH3), can translocate to the nucleus, where they ultimately generate αKG [30], although to date this has only been demonstrated during a time-limited phase of zygotic genome activation. The generation and presence of TCA cycle metabolites in multiple cellular compartments increase the chance of them being able to interact with a number of enzymes, and this will be discussed later in this review.

Whilst the standard model of the TCA cycle describes a unidirectional process, the landscape is more complex than this. Instead of OAA condensing with acetyl-CoA to form citrate, it can instead be converted to malate, then fumarate, and back to malate and OAA, in a process termed ‘backflux’ [34]. Truncation of the TCA cycle is also possible in certain disease states, including hypoglycaemia and in various cancers, in which specific steps are no longer functional and are bypassed. During hypoglycaemia, for example, glutamate can become the primary source of carbon, allowing OAA to be generated from αKG instead of citrate [35]. Loss of FH function, as found in some cancers, results in the accumulation of fumarate and a corresponding decrease in NADH generation [36]. However, despite these metabolic alterations fumarate hydratase-deficient cells (UOK262 cell line) are viable, and resist hypoxia, perhaps through stabilisation of hypoxia-inducible factors [36], which will be discussed later in this review. The plasticity of the TCA cycle serves to illustrate that it can undergo complex adaptations in pathological states and promote cell survival.

Hepatic lipid accumulation is associated with mitochondrial dysfunction and oxidative stress, through disruption of mitochondrial energy metabolism pathways [17, 37–39]. Studies in human and animal models show that the transport of excess FFAs into the mitochondria leads to compensatory adaptations, which increase the rates of β-oxidation, TCA cycle flux, and oxidative phosphorylation [23, 40, 41]. These alterations are associated with increased reactive oxygen species (ROS) generation, lipid peroxidation, and apoptosis [42], which may contribute to the inflammatory phenotype observed in patients with NASH. Humans with NASH demonstrate increased expression of mitochondrial uncoupling protein 2 (UCP2), which may occur in response to increased ROS production [43]. UCP2 uncouples the electron transport chain, by increasing proton leakage and lowering redox stress through decreased reactive ROS production [44]. Whilst this may initially be beneficial, it may eventually become detrimental in chronic conditions such as NAFLD, leading to mitochondrial dysfunction, with loss of electron transport chain efficiency, decreased production of adenosine triphosphate (ATP), and increased ROS generation [45, 46].

Evidence from studies in humans and in mouse models suggests that TCA cycle activity is increased in NAFLD, with increased cycle flux and replenishment (anaplerosis) of αKG via glutamate. Although the evidence for this is compelling, the technical difficulties of obtaining human tissue samples mean that many of the changes in hepatic TCA cycle flux and anaplerosis which have been described in human studies have been inferred from peripheral blood, and thus the results may be confounded by the release of metabolites from other tissues [23]. Although there are limited data from humans with NASH suggesting that αKG is decreased in diseased hepatic tissue [47], the tissue samples were first embedded in paraffin with downstream metabolomic analysis which may confound some of the results [48], so that further studies would be useful to confirm these findings. Nuclear magnetic resonance (NMR) studies also indicate increased hepatic TCA cycle flux and anaplerosis in human NAFLD [23] and studies in the livers of mice fed a high-fat diet demonstrate increased TCA cycle flux associated with hepatic steatosis [49]. The use of whole tissue to examine the effects of NAFLD on metabolic function can make it difficult to determine which cells within a tissue are exhibiting altered TCA cycle activity. However, a recently developed in vitro model, which uses embryonic stem cell (ESC)-derived hepatocytes to model NAFLD, supports the assertion that intracellular lipid accumulation associates with dysregulation of the TCA cycle and altered abundance of intermediates [50].

## The αKG-dependent dioxygenases

In addition to its central role in energy metabolism, components of the TCA cycle can influence the catalytic activity of the αKG-dependent dioxygenase family of enzymes [51]. The αKG-dependent dioxygenases are Fe(II)/O_2_-dependent enzymes, which catalyse the hydroxylation of a number of substrates, including DNA, histones, and proteins. The enzyme family includes the prolyl-4-hydroxylases (PHD), Jumonji domain-containing histone demethylases (JHDMs), and ten-eleven translocation (TET) dioxygenases [52]. All of the enzymes within this family have two binding domains in their β-sheet core, which are highly specific for αKG, and binding of αKG acts as a positive regulator of dioxygenase activity [53, 54]. Conversely, these two domains can be competitively bound by succinate and fumarate, which inhibit dioxygenase activity [55]

The functions of these enzyme members are numerous but include epigenetic regulation, through DNA methylation, histone methylation, and acetylation, as well as oxygen sensing through the PHDs [56–58]. It is tempting to speculate, therefore, that the αKG-dependent dioxygenases could form a nexus, integrating environmental signals and disseminating them to downstream targets by altering gene transcription to allow cells to maintain homeostasis. Thus, dysfunctional mitochondrial (TCA cycle) metabolism could impact on epigenetic modifications of DNA and chromatin through alterations in JHDM, PHD, and TET enzyme activity, and findings from human breast and colon cancer cell lines open up the possibility that this may also play a role in NAFLD [59–61].

## Prolyl-4-hydroxylases, HIF-1α, and NAFLD

**Fig. 2:**
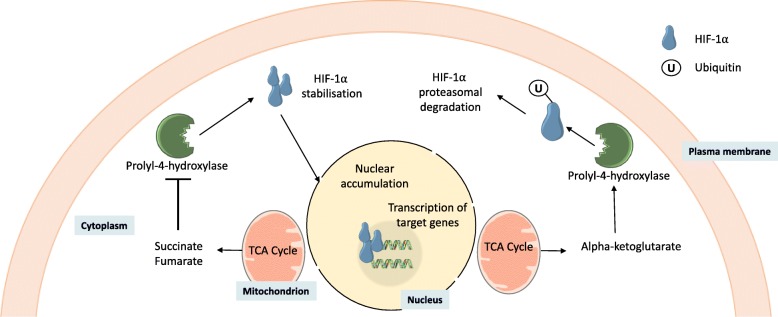
TCA cycle metabolites in the cytoplasm can interact with prolyl-4-hydroxylases, to either promote or repress activity. Repression prevents ubiquitination of HIF-1α, promoting protein stabilisation, followed by nuclear accumulation and transcription of target genes

PHDs catalyse the most prevalent post-translational modification (PTM) in humans, hydroxylating proline residues to form (2S,4R)-4-hydroxyproline [62]. Whilst PHDs have many substrates, including Argonaut 2 [63], collagen [64], and elastin [65], one of the most well characterised are the hypoxia-inducible factor (HIF) transcription factors, which are targeted by PHD2 [66]. HIF-1 is comprised of two constitutively expressed sub-units: HIF-1α and HIF-1β [62]. Under normal oxygen conditions (normoxia), PHDs hydroxylate two proline residues (Pro402 and Pro564) within the oxygen-dependent degradation domain of HIF-1α [62]. The hydroxylation of these proline residues recruits ubiquitin E3 ligase and leads to the polyubiquitination of HIF-1α, marking it for degradation by the proteasome [67]. Under hypoxic conditions, low oxygen concentrations diminish the ability of PHDs to hydroxylate these proline residues, and thus HIF-1α stabilises, allowing it to translocate to the nucleus where it can dimerise with HIF-1β, bind to hypoxia response elements, and facilitate gene transcription [68] (Fig. 2).

Despite their name, it is not only oxygen that regulates the HIFs. Since PHDs are also regulated by succinate, fumarate, and αKG, HIFs are able to coordinate gene networks in response to metabolic rewiring [69, 70]. Indeed, in human breast cancer cell lines, hypoxia is associated with lipid homeostasis, through coordination of genes regulating lipid droplet formation, de novo lipogenesis, and fatty acid uptake [71]. Whilst there is a lack of data to directly link TCA cycle dysfunction in NAFLD with PHD inhibition and HIF stabilisation, there are limited data from rat models demonstrating altered HIF-1α mRNA and protein levels in steatotic whole liver [72]. A caveat to this study is that it was performed in whole tissue, and perivenous regions of the liver express higher levels of HIF mRNA [73] so that disease-associated alterations in hepatic structure and/or variation in the region of the liver sampled could influence the results. However, in support of this link, studies suggest that there is a relationship between lipid accumulation and HIF-1α stabilisation [74]. Both HIF-1α mRNA and protein levels increase in the adipose tissue of obese subjects, and there is strong evidence that obesity provokes a hypoxia-like response in adipose tissue [75, 76]. Tracer studies in mice, using a ^13^C-glucose stable isotope, demonstrated an increase in the abundance of αKG, succinate, and fumarate in the adipose tissue of obese animals [24]. Furthermore, this study found increased levels of succinate in the liver of obese mice, suggesting that obesity is associated with alterations in metabolites that have the potential to impact on the activity of PHDs [24].

There are conflicting reports as to whether HIF-1α plays a protective or harmful role in NAFLD [72, 77]. Using a rat model of liver steatosis, Carabelli et al. describe increased expression of HIF-1α mRNA in the liver and suggest that this is responsible for an observed increase in mitochondrial biogenesis [72]. They also speculate that this may form a protective mechanism, whereby hepatocytes are able to increase rates of β-oxidation to process excess intracellular lipids [72]. In contrast, Mesarwi et al. observed that HIF-1α mediates the development of hepatic fibrosis in a mouse model of NAFLD, suggesting a pathogenic role [77]. The discrepancies between these studies could be explained by the use of different animal models. It may also be a result of NAFLD comprising a spectrum of pathology, whereby some molecular mechanisms may be protective during the early stage of disease, but eventually become chronic and harmful. However, there is a lack of data to suggest whether HIF-1α is important for the development of NAFLD in humans and for any impact of TCA cycle dysfunction on PHD catalytic activity. Nevertheless, PHD2 contains a histone-binding domain [78], can translocate to the nucleus, and is found within the chromatin fraction [62], suggesting that it may directly impact on gene transcription.

## JHDMs, histone modifications, and NAFLD

Histone proteins form cores around which DNA can condense, allowing it to be packaged efficiently into nucleosomes, to form chromatin [79]. These nucleosomes can be highly compacted, preventing the transcription of DNA to mRNA [80]. Histones are also subject to many PTMs, including acetylation, methylation, phosphorylation, sumoylation, and ubiquitylation [81]. Depending upon the deposition site of PTMs, they can result in chromatin condensation and transcriptional silencing [82] or associate with relaxation of chromatin and active gene transcription [82].

The JHDMs are a large family of αKG-dependent dioxygenases that can catalyse the removal of mono-, di-, and trimethylation marks from specific lysine (K) residues of histone 3 (H3) or histone 4 (H4) [83, 84]. Each JHDM targets a specific histone lysine residue, which impacts on chromatin structure in multiple ways, depending on the resulting methylation pattern. Broadly, methylation of H3K9 and H3K27 is associated with chromatin compaction and transcriptional silencing [85]. In contrast, H3K4 and H3K36 methylation are typically associated with chromatin relaxation and transcriptional activity [85]. However, enrichments of H3K9 di- and trimethylation have been observed in transcriptionally active euchromatic regions of genes [86], demonstrating that the impact of histone methylation on gene transcription is complex and nuanced.

The activity of the JHDMs can be influenced by TCA cycle metabolites, for example, the experimental silencing of succinate dehydrogenase in a human HCC cell line leads to the accumulation of succinate, with resultant inhibition of the JHDMs, altered histone methylation patterns, and changes in transcriptional activity [87]. Although there is no direct evidence to link TCA cycle dysfunction with alterations in JHDM activity in NAFLD, in a mouse model, hepatic steatosis occurred in association with altered JHDM expression and altered H3K4 and H3K9 trimethylation at the peroxisome proliferator-activated receptor alpha (PPARα) gene and in a number of other genes important in lipid catabolism [88].

## DNA methylation and NAFLD

**Fig. 3:**
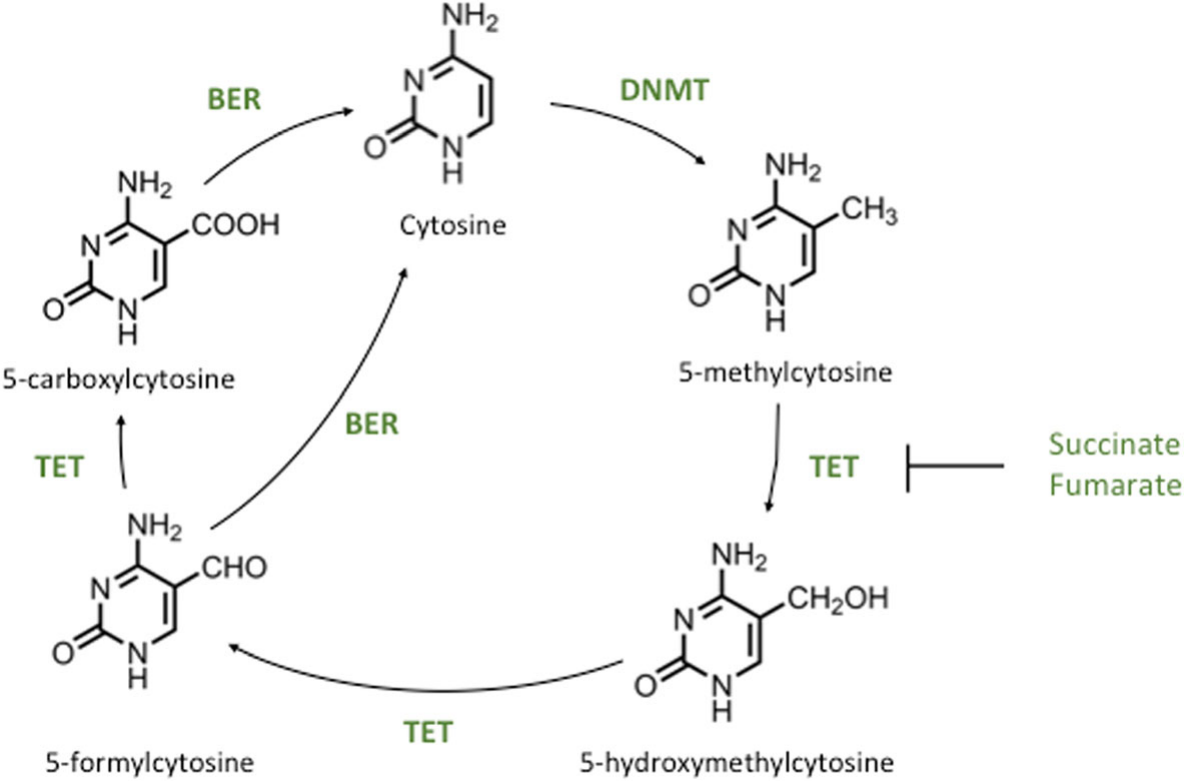
TET1 oxidation of 5mC through to unmodified cytosine. After the deposition of 5-methylcytosine by the DNMT enzymes it can be oxidised by the TETs to 5-hydroxymethylcytosine, 5-formylcytosine, and then 5-carboxylcytosine, before base excision repair resulting in unmodified cytosine. These iterative oxidation steps can be inhibited by the TCA cycle metabolites succinate and fumarate

DNA methylation is a highly conserved epigenetic modification involving the addition of a methyl group at the C5 position of cytosine, predominantly in cytosine-phosphate-guanine (CpG) dinucleotides, to form 5-methylcytosine (5mC) [89]. The reactions are catalysed by the DNA methyltransferases (DNMTs). Approximately 10% of CpG sites are located in clusters termed CpG islands (CGIs) and are most commonly found in the promoters of genes with important developmental functions [90]. In general, these CGIs are unmethylated, but where methylation does occur, it can be associated with transcriptional repression and heterochromatic regions, as 5mC is, in general, thought to prevent transcription factor binding [91]. During development, it is estimated that fewer than 30% of CpGs are methylated, in contrast to 85% in terminally differentiated cells, illustrating the tight control that is exerted to retain cell homeostasis and phenotype in differentiated cells [92]. The TET enzymes are a subset of the αKG-dependent dioxygenase family, comprised of TET1, TET2, and TET3. These enzymes catalyse the iterative oxidation and demethylation of 5mC to 5-hydroxymethylcytosine (5hmC), 5-formylcytosine (5fC), and 5-carboxylcytosine (5caC), before base excision repair (BER) is triggered, resulting in unmodified cytosine [56] (Fig. 3). To achieve this, the double-stranded β-helix at the N-terminus of the TET enzymes brings together Fe(II), αKG, and 5mC to allow oxidation of the methyl group to 5hmC, 5fC, and 5caC [56]. The contiguous cysteine-rich domain then wraps around the double-stranded β-helix to stabilise the interaction between the TET enzyme and the DNA, facilitating the oxidation reaction [56]. Whilst initially thought to be transient, 5hmC is now accepted as a stable cytosine modification [93], and 5hmC enrichment within gene bodies and enhancers broadly correlates with transcriptional activation [94, 95]. In contrast, 5hmC enrichment within gene transcriptional start sites (TSS) is associated with transcriptional repression and the maintenance of CpG hypomethylation [96]. As with other enzymes in the αKG-dependent dioxygenase family, TET catalytic activity is modulated by the TCA cycle metabolites αKG, succinate, and fumarate [55]. This may be important in some human cancers, for example, loss of function mutations in fumarate hydratase and succinate dehydrogenase in human cervical cancer and glioblastoma cell lines result in the accumulation of fumarate and succinate, respectively, with consequent inhibition of TET enzyme activity and altered 5hmC levels [55].

The development of hepatic steatosis is associated with changes in hepatic DNA methylation in a methionine-deficient diet (MDD) model of NAFLD in mice [97]. In this study, it is not known where in the genome these changes occur, making it difficult to infer any impact on transcriptional regulation. Furthermore, there are concerns as to how well the MDD model recapitulates NAFLD, as shown by suppressed expression of very low-density lipoprotein carboxylesterase mRNA [98]. In contrast, Pirola et al. observed that humans with NASH have increased DNMT1 mRNA and a concurrent increase in DNA methylation of the mitochondrial NADH dehydrogenase 6 gene in the whole liver [99]. The importance of mitochondrial DNA methylation is debated [100, 101], and it is therefore currently uncertain what impact it may have on the pathogenesis of NAFLD.

Evidence of altered nuclear DNA methylation in the liver in NAFLD is supported by a number of studies in humans. Differential methylation has been reported in liver biopsy specimens from patients with NAFLD [102, 103], with some studies showing an inverse correlation between gene expression and DNA methylation [104]. Specific differentially methylated regions have been identified which may associate with NAFLD progression [105]. Notably, some of the changes in DNA methylation may be reversible with weight loss, for example following bariatric surgery [104, 105]. There is also evidence from bariatric surgery patients suggesting that there is a correlation between altered DNA methylation and changes in hepatic insulin signalling [106]. A recent study focused on understanding the link between NAFLD-associated liver fibrosis and cirrhosis and DNA methylation performed bisulfite sequencing in liver biopsies from female patients undergoing bariatric surgery, who had no other liver-related pathologies [107]. The study identified differential CpG island methylation at a number of different genes and gene ontology analysis showed enrichment for a number of pathways related to NAFLD, including reactive oxygen species production and T2D signalling [107]. Whilst compelling, the findings in each of these studies are correlative and do not describe any mechanism(s) by which altered DNA methylation might occur. In addition, the studies used bisulfite treatment to analyse DNA methylation, making it impossible to determine if they were observing changes in 5mC or its oxidised counterpart 5hmC.

There is evidence to suggest that 5hmC levels are altered in NAFLD. Patient studies describe a positive association between global hepatic 5hmC levels and the expression of peroxisome proliferator-activated receptor γ (PPARγ) coactivator 1α (PPARGC1A), a gene at which DNA methylation has previously been associated with NAFLD and IR [99, 108]. The lack of any change in global 5hmC could reflect the use of whole tissue, which may dilute the 5hmC signal through analysis of multiple cell types or alternatively may indicate that alterations in 5hmC in response to NAFLD occur only at specific genes. Indeed, using an in vitro model of NAFLD, we have recently shown that changes in 5hmC levels occur specifically at induced genes associated with lipid synthesis and transport [50]. Further, we found altered expression of several genes encoding TCA cycle enzymes, including succinate dehydrogenase, isocitrate dehydrogenase, and an accumulation of the TCA cycle metabolite oxalosuccinate, suggesting altered cycle activity. The importance of the TET enzymes and 5hmC in liver disease is further supported by studies showing that aberrant TET1 activity in hepatocellular carcinoma is associated with loss of 5hmC and a gain of 5mC, with subsequent transcriptional repression, suggesting that the TETs may normally prevent DNMT binding in these regions in the healthy liver [109].

## TET1 and histone methylation

In human ES cells, TET1 may orchestrate the activity of components of the epigenetic machinery by targeting genes for binding by the polycomb recessive complex (PRC). Though they do not appear to physically interact, TET1 appears to recruit PRC2 to particular genes [110], possibly by maintaining a hypomethylated state, as DNA methylation prevents binding by PRC2 [111]. Upon binding to hypomethylated DNA, PRC2 can di- or trimethylate H3K27, an epigenetic mark associated with transcriptional repression [112, 113]. This may be relevant to the pathogenesis of NAFLD, since genome-wide association studies have shown enrichment of PRC2 pathways in humans with NASH [114]. In support of this, data from rat liver and human liver cell lines have demonstrated that downregulation of core components of PRC2 is associated with the de-repression of inflammatory genes and intracellular lipid accumulation [115]. Thus, whilst TET1 may not directly control the DNA-binding activity of PRC2 in the pathogenesis of NAFLD, these data suggest that it may facilitate it.

## Post-translational regulation of TET activity

In addition to regulation by TCA cycle metabolites, Fe(II), and oxygen, the TET enzymes are also regulated by PTMs, including phosphorylation and *O*-GlcNAcylation. The *O*-GlcNAcylation PTM is deposited by *O*-linked *N*-acetylglucosamine transferase (OGT), which attaches an *N*-acetyl glucosamine to serine and threonine residues within target proteins [116]. Disruption of the OGT binding site in TET1 reduces genomic 5hmC levels, with a concurrent reduction in gene expression [117]. The post-translational addition of *O*-GlcNAcylation to proteins is promoted by the hexosamine biosynthetic pathway (HBP), which synthesises *N*-GlcNAc from glucose, linking elevated intracellular glucose to increased *O*-GlcNAcylation [118]. This generates a positive feedback loop, whereby the HBP diverts glucose away from glycolysis, and lowers αKG levels. This, in turn, promotes stabilisation of HIF-1α via PHD inhibition and promotes increased transcription of the glucose transporter 1 (GLUT1) gene [119], allowing more glucose to enter the cell. The reduction of αKG in response to elevated *O*-GlcNAcylation could indicate that, whilst TCA cycle intermediates play a direct role in TET enzyme function, they may also play an indirect role through the modulation of PTMs. Indeed, the impact of increased HBP activity, subsequent stabilisation of HIF-1α, and ensuing increase in intracellular glucose may be more important for directing the activity of the TETs. This is relevant, since NAFLD is strongly associated with T2D, hepatic IR, and poor glucose clearance [120]. In addition, studies in mouse models and human tissue show that OGT also promotes the transition from hepatic steatosis to HCC [121]. Whilst this effect is thought to be mediated by palmitic acid production and endoplasmic reticulum stress, other avenues have not yet been explored. In mouse ES cells, OGT can form a complex with TET1 or TET2 in the nucleus, anchoring them to histones and DNA [122], and since genomic hypomethylation is a hallmark of HCCs [123], it is possible that this complex may be involved in its development. This adds to the wealth of evidence suggesting that the TET enzymes and 5hmC may play a role at all stages of NAFLD pathogenesis, and its progression to HCC.

## Future perspectives

The study of epigenetic dysregulation in disease is fraught with difficulties and limitations, not least whether changes in epigenetic marks are a cause or consequence of the disease process itself [124]. In addition to this, many studies in the NAFLD field have reported changes in genome methylation, but have arrived at their conclusions based on bisulfite sequencing data [99, 104]. Since bisulfite sequencing cannot distinguish between 5mC and 5hmC [125], it is impossible to determine precisely which changes are occurring during the pathogenesis of NAFLD in much of the current literature. Further to these difficulties are the inherent limitations of the current models of NAFLD, which make it difficult to study the interplay between the TCA cycle and epigenetic modifications. It is technically difficult, and highly invasive, to obtain liver samples from humans, and controls are often not taken from healthy donors but individuals being investigated for pathologies other than NAFLD [104]. The use of whole tissue samples from humans or animals makes it challenging to attribute phenotypes to a particular cell type. This could, typically, be overcome through the use of fluorescence activated cell sorting, but recent studies have shown that this alters the redox status of cells [126], potentially confounding analyses of changes in the TCA cycle. The use of HCC cell lines is also challenging, since malignant transformation often results in metabolic derangement [127]. The development and refinement of 2-D and 3-D in vitro models of ‘NAFLD in a dish’ using a single cell type may be useful for future mechanistic and therapeutic studies, including the careful dissection of the interplay between TCA cycle dysfunction and epigenetic regulation of gene expression in the pathogenesis of the disease [50, 128]

## Conclusions

As the obesity pandemic continues, the number of people living with NAFLD will continue to grow, increasing the economic burden on healthcare systems globally [129], making it critical that new strategies for the treatment of the disease are developed. Studies of the interplay between TCA cycle dysfunction and regulation of the αKG-dependent dioxygenases in new models may provide a more comprehensive picture of the processes underlying NAFLD and lead to the development of predictive tests or new diagnostics, alongside novel therapeutics, which are able to reverse the disease.

## Data Availability

No new data/materials are contained in the manuscript.

## Abbreviations

5caC: 5-carboxylcytosine
5fC: 5-formylcytosine
5hmC: 5-hydroxymethylcytosine
ATP: Adenosine triphosphate
BER: Base excision repair
CGI: CpG island
CpG: Cytosine-phosphate-guanine
DNMT: DNA methyltransferase
ESC: Embryonic stem cell
FFA: Free fatty acids
FOXO1: Fork-head box protein
GLUT1: Glucose transporter 1
GLUT4: Glucose transporter 4
H3: Histone 3
H4: Histone 4
HBP: Hexosamine biosynthetic pathway
HCC: Hepatocellular carcinoma
HIF: Hypoxia-inducible factor
IDH: Isocitrate dehydrogenase
IDH3: Isocitrate dehydrogenase 3
IR: Insulin resistance
JHDM: Jumonji domain-containing histone demethylase
MDD: Methionine-deficient diet
MDH: Malate dehydrogenase
NAFLD: Non-alcoholic fatty liver disease
NASH: Non-alcoholic steatohepatitis
NMR: Nuclear magnetic resonance
OAA: Oxaloacetate
OGT: *O*-linked *N*-acetylglucosamine transferase
PC: Pyruvate carboxylase
PDH: Pyruvate dehydrogenase
PHD: Prolyl-4-hydroxylase
PPARGC1A: PPARγ coactivator 1α
PPARα: Peroxisome proliferator-activated receptor alpha
PPARγ: peroxisome proliferator-activated receptor γ
PRC: Polycomb recessive complex
PTM: Post-translational modification
ROS: Reactive oxygen species
T2D: Type 2 diabetes
TCA: Tricarboxylic acid
TET: Ten-eleven translocation dioxygenases
UCP2: Uncoupling protein 2
αKG: Alpha-ketoglutarate
